# The Expression of Three Opsin Genes from the Compound Eye of *Helicoverpa armigera* (Lepidoptera: Noctuidae) Is Regulated by a Circadian Clock, Light Conditions and Nutritional Status

**DOI:** 10.1371/journal.pone.0111683

**Published:** 2014-10-29

**Authors:** Shuo Yan, Jialin Zhu, Weilong Zhu, Xinfang Zhang, Zhen Li, Xiaoxia Liu, Qingwen Zhang

**Affiliations:** 1 Department of Entomology, China Agricultural University, Beijing, P.R. China; 2 Beijing Entry-Exit Inspection and Quarantine Bureau, Beijing, P.R. China; Karlsruhe Institute of Technology, Germany

## Abstract

Visual genes may become inactive in species that inhabit poor light environments, and the function and regulation of opsin components in nocturnal moths are interesting topics. In this study, we cloned the ultraviolet (UV), blue (BL) and long-wavelength-sensitive (LW) opsin genes from the compound eye of the cotton bollworm and then measured their mRNA levels using quantitative real-time PCR. The mRNA levels fluctuated over a daily cycle, which might be an adaptation of a nocturnal lifestyle, and were dependent on a circadian clock. Cycling of opsin mRNA levels was disturbed by constant light or constant darkness, and the UV opsin gene was up-regulated after light exposure. Furthermore, the opsin genes tended to be down-regulated upon starvation. Thus, this study illustrates that opsin gene expression is determined by multiple endogenous and exogenous factors and is adapted to the need for nocturnal vision, suggesting that color vision may play an important role in the sensory ecology of nocturnal moths.

## Introduction

Vision is one of the most familiar forms of stimulus discrimination and plays numerous key roles in the performance of insect behaviors, such as searching for food and potential mates, avoiding predators and unsafe environments, and other specific behaviors [1]–[5]. In Lepidoptera, visual information is acquired via a highly developed visual system. Compound eyes are usually composed of several thousand ommatidia to detect and convert light into visual images, and each ommatidium contains nine photoreceptor cells [6]–[10]. At the molecular level, the visual pigments in photoreceptors include a vitamin A-derived chromophore, usually 11-*cis*-retinal, and a transmembrane protein, opsin [11]–[12]. The amino acid sequences of both the opsin and the chromophore affect their ability to absorb visual pigment [12]–[14]. Opsins are ancient proteins belonging to the G-protein-coupled receptor family and are characterized by a seven-transmembrane domain structure and a lysine residue in the seventh helix [15]–[17]. There are three types of opsins with peak absorbance at either the ultraviolet wavelengths (UV, 300–400 nm), blue wavelengths (BL, 400–500 nm) or long wavelengths (LW, 500–600 nm) [8], [10], [12].

Regulation of opsin mRNA levels has been studied in several species. Daily changes in opsin mRNA levels are typically observed in animals, including mice [18], fish [19], toads [19], rats [20]–[21], honeybees [22] and horseshoe crabs [23]–[25]. All living organisms possess a circadian clock to synchronize their rhythm with the environment, and photoreceptors are clearly necessary for this synchronization [26]–[28]. These fluctuations in opsin mRNA levels are regulated by an endogenous circadian clock and underlie the ability of the visual system to function optimally in ambient illumination [19], [21]–[25]. Furthermore, opsin mRNA is induced by light exposure, and organisms exposed to more light exhibit elevated opsin gene expression [14], [22], [29]–[32]. Sexual dimorphism in the expression pattern of opsin genes has also been observed in previous studies [33]–[34], and sexually dimorphic photoreceptors, which are adapted to differences in the behaviors of males and females, are common in butterflies [35]–[37]. Thus, regulation of opsin mRNA may be controlled by multiple endogenous and exogenous factors.

The role of photoreceptors in Lepidoptera is an interesting topic because of the wide variation in habits and lifestyle. Most studies on the regulation of opsin mRNA have been performed in diurnal animals, whereas almost no information exists on opsin regulation in nocturnal animals. Whether the factors influencing opsin mRNA expression are similar between diurnal and nocturnal insects remains unknown. There have been several studies on electroretinography (ERG), phototaxis, circadian rhythms and sexual behavior in the cotton bollworm [38]–[42], making this organism not only a worldwide pest responsible for great economic losses but also an excellent model for studying the evolution and function of opsins. Xu *et al.* [32] found no daily cycling of opsin mRNA levels in *H. armigera*. In that study, the authors collected RNA samples from whole individual moths, but we think it is more important to evaluate opsin expression patterns specifically in the compound eye. In the current study, we focused on selected genes encoding opsins in *Helicoverpa armigera* (Hübner) and investigated the determinants of opsin mRNA levels in a specific tissue (the compound eye), which was beneficial for improved understanding of the evolution and function of opsins in nocturnal moths.

## Materials and Methods

The wording of our manuscript is suitable for publication. Our study was conducted in the IPM lab at China Agricultural University (40°02′N, 116°28′E). The living material sampled in our experiments consisted of cotton bollworms (*Helicoverpa armigera*). No specific permits were required for the insect collection performed for this study. Larvae of *H. armigera* collected from a cotton field in Hebei Province (China) were used in the experiments. Cotton bollworms are a common insect and are not included in the “List of Protected Animals in China”.

### 1. Animals and experimental conditions

Larvae of *H. armigera* collected from a cotton field in Hebei Province (China) were used in the experiments. The larvae were reared on a synthetic diet [43] and maintained at 27±1°C and 75±10% relative humidity (RH) with a 14∶10 light:dark photoperiod. Zeitgeber time 0 (ZT0) was designed as lights-on, and ZT14 was designed as lights-off. Pupae were segregated by sex, placed in holding cages (20×25×30 cm) with a removable white cloth top for egg collection, and held for adult emergence. The moths were provided a 10% honey solution. Moths that emerged during the scotophase were designated as 0-day-old, 1-day-old, 2-day-old, and so forth on subsequent days.

### 2. Isolation and cloning of opsin-encoding cDNA

Total RNA was isolated from the compound eye of 2-day-old moths using the RNeasy Mini Kit (Qiagen, Hilden, Germany), treated with DNase I (Qiagen) to remove any residual genomic DNA, and pelleted twice by successive centrifugation through 5.7 mol/L CsCl according to Chase *et al.* [13]. A 1 µL sample of RNA was used to perform spectroscopic quantitation using a NanoDrop 2000 spectrophotometer (Thermo Fisher, USA). Then, 2 µg samples of total RNA were reverse-transcribed using M-MLV Reverse Transcriptase (Promega, USA).

Several primers (Table S1) were used to amplify fragments of the UV-sensitive opsin gene via polymerase chain reaction (PCR). Because of the conserved 5′ untranslated regions of the blue- and long wavelength-sensitive opsin genes, primers (Table S1) were designed to clone the 5′ fragments. PCR reactions were typically cycled 35 times at 94°C for 45 s, 55°C for 30 s, and 72°C for 1 min. To obtain complete sequences of the three opsin genes, rapid amplification of cDNA ends was performed using the FirstChoice RLM-RACE Kit (Ambion, Austin, TX). The amplification protocols consisted of 35 cycles of 94°C for 45 s, 58°C for 30 s, and 72°C for 90 s. TransTaq-T DNA Polymerase (TransGen Biotech, Beijing, China) was used to perform the PCR reactions. The purified fragments were cloned into the Trans1-T1 vector (TransGen Biotech) and then transformed into DH5α *Escherichia coli*; positive clones were then selected for sequencing (BGI Life Tech Co., Beijing, China).

### 3. Sequence analysis and construction of the phylogenetic tree

The opsin gene sequences were submitted to the NCBI website (http://www.ncbi.nlm.nih.gov), and translations of the opsin genes were performed using DNAMAN v.5.2.2 (Lynnon Biosoft, Quebec, Canada). Predictions of the transmembrane regions were made using TMpred (http://www.ch.embnet.org/software/TMPRED_form.html) [44]. The theoretical isoelectric points (pI) and molecular weights (MW) were calculated using the Compute pI/Mw tool (http://web.expasy.org/compute_pi/). The protein functional sites were predicted using PROSITE SCAN (http://npsa-pbil.ibcp.fr/cgi-bin/npsa_automat.pl?page=/NPSA/npsa_proscan.html) [45]–[46]. All opsin sequences, including those obtained from the present study, were retrieved from GenBank (Table S2). The phylogenetic tree was constructed using the neighbor-joining method in MEGA 4.0.

### 4. Validation of opsin gene expression using quantitative real-time PCR (qRT-PCR)

**Tissue specificity:** We extracted total RNA from various tissues (compound eye, brain, antennae, legs, thorax, abdomen, and wings) of 2-day-old moths at zeitgeber time 1 (ZT1). The entire brain was harvested. The head capsule was opened, and the tissues covering the brain were removed. Total RNA was isolated from the compound eyes of 2-day-old moths for the following tests. **Rhythmicity with photoperiod:** RNA samples were collected at 2 h intervals (ZT1, ZT3, ZT5, ZT7, ZT9, ZT11, ZT13, ZT15, ZT17, ZT19, ZT21 and ZT23, which correspond to CT1 (circadian time 1), CT3, CT5, CT7, CT9, CT11, CT13, CT15, CT17, CT19, CT21 and CT23) from the following moths. (1) Moths kept under 14L:10D, constant darkness (DD) and constant light (LL) (*H. armigera* were entrained for 7 days under 14L:10D, DD and LL). *H. armigera* reared under 14L:10D were transferred to constant DD and LL, and 2-day-old moths reared under constant DD and LL were collected according to the duration of actual clock hours. (2) Moths kept under 14L:10D and then transferred to DD (*H. armigera* were entrained for 1 day under DD). **Exposure to various wavelengths of light:** 500-lux light of three wavelengths, including UV (mainly 365 nm), blue (mainly 450 nm), and green (mainly 505 nm), using a light irradiation system made by FSL, Foshan, China were used instead of scotophase, and RNA samples were collected after 6 h of radiation at ZT20. **Starvation:** RNA was extracted from moths that were not fed after adult emergence at ZT1. **Copulation:** Twenty pairs of virgin moths were paired for mating in 20×25×30 cm cages, which were checked every 15 min throughout scotophase to determine whether copulation had occurred. We then obtained RNA samples at 0 h and 3 h after moth copulation.

The circadian clock genes *cryptochrome1* and *cryptochrome2* [40] were assayed in this study. qRT-PCR was carried out on the ABI 7300 instrument (ABI, Ambion) in reactions containing 5 µl of SybrGreen (ABI, Ambion), 100 nM of forward and reverse primers, 1 µl of cDNA from the three opsin genes and 5 µl of cDNA from the circadian clock genes. The amplification protocols consisted of an initial denaturation step at 95°C for 10 min; 40 cycles of 94°C for 15 s, 55°C for 40 s, and 72°C for 35 s; and a melting curve ramp to confirm that each reaction did not produce nonspecific amplification. *EF-1α* and *RPS15* were used as the reference genes [14], [22], [40], [47], and the amount of transcript from each gene was normalized to the abundance of *EF-1α* and *RPS15* using the 2^−ΔΔCt^ method described by Livak and Schmittgen [48]. Technical assays were carried out independently and in triplicate per cDNA sample, and all results were obtained from three independent RNA samples.

### 5. Statistical analysis

All statistical analyses were conducted using the SPSS 16.0 software (IBM, Armonk, NY). Differences in gene expression between two experimental treatments were examined using independent t-tests. Other data were analyzed using one-way ANOVA with the Tukey HSD test when the data were homoscedastic or the Games-Howell test when the data were not. In all tests, *P* values<0.05 were considered significant.

## Results

### 1. Cloning and characterization of three opsin genes in cotton bollworms

We have cloned three opsin genes from the compound eyes of *H. armigera*: UV-, blue-, and long-wavelength-sensitive opsin genes, which are designated “*Ha-UV*,” “*Ha-BL*,” and “*Ha-LW*,” respectively. Including the 5′ and 3′ UTRs, 1258 bp of *Ha-UV*, 1451 bp of *Ha-BL*, and 1574 bp of *Ha-LW* were cloned and sequenced. These three cDNAs encoded opsins of varying lengths: 379 (Ha-UV), 381 (Ha-LW) and 382 (Ha-BL) amino acids. It is possible that the full 3′ untranslated sequences of *Ha-UV*, *Ha-BL*, and *Ha-LW* were not cloned because we could not identify a polyadenylation signal with the sequence AATAAA. The molecular masses of the encoded opsins were predicted to be 41.35 kDa (Ha-UV), 43.22 kDa (Ha-BL), and 41.86 kDa (Ha-LW), and the calculated isoelectric points (pI) were 8.05 (Ha-UV), 7.02 (Ha-BL), and 7.97 (Ha-LW). The encoded amino acid sequences of *H. armigera* opsins exhibited various conserved characteristics compared with opsins in general or with insect-specific opsins, including seven membrane-spanning helical domains, visual pigment (opsin) retinal binding sites and G-protein-coupled receptors (Figure S1). *H. armigera* opsins shared putative posttranslational modification sites, including N-myristoylation sites, protein kinase C phosphorylation sites, casein kinase II phosphorylation sites, and N-glycosylation sites.

### 2. Sequence comparison and phylogenetic analysis

**Figure 1:**
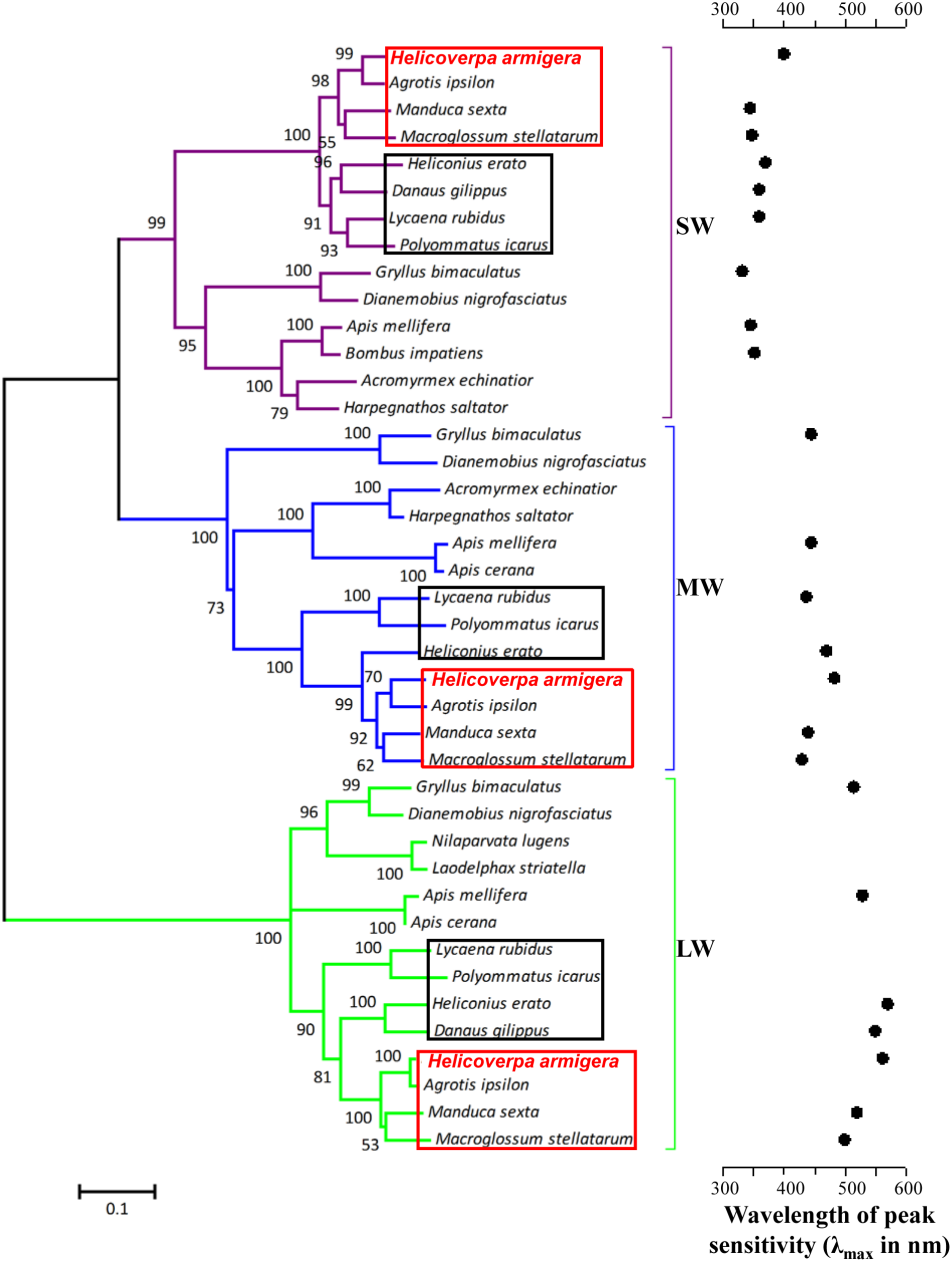
Evolutionary origin of opsins in *H. armigera*. The phylogenetic tree is based on aligned and full-length amino acid sequences. Numbers at the nodes indicate the bootstrap, and all nodes are supported by more than 50%. The various insect opsin lineages are colored in purple (SW = short-wavelength clade), blue (MW = middle-wavelength clade) and green (LW = long-wavelength clade). The wavelength of peak sensitivity (λ_max_) of the respective visual pigment is given [12], [39], [69]. *H. armigera* is highlighted in red and bold. Moths and butterflies are highlighted in red and black boxes, respectively.

We reconstructed a molecular phylogenetic tree of insect visual opsins to clarify their evolutionary origin (Figure 1). The *H. armigera* sequences clustered in the visual opsin clades of insects: the short-wavelength (SW), middle-wavelength (MW) and long-wavelength (LW) branches. The results of the phylogenetic analysis agreed with the structure and distribution of these opsins. Moreover, the phylogenetic tree demonstrated that SW opsins exhibited a shorter genetic distance to MW opsins than to LW opsins. Based on the spectral sensitivities of compound eyes, the SW, MW and LW opsins were assigned to the UV, blue and green opsin genes, respectively. Sequence alignment revealed that *H. armigera* opsins shared significant homology with opsins identified from other insects, such as *Manduca sexta* (UV: 88% identity; blue: 90% identity; green: 90% identity), *Lycaena rubidus* (UV: 84% identity; blue: 72% identity; green: 77% identity), *Gryllus bimaculatus* (UV: 57% identity; blue: 58% identity; green: 76% identity), and *Apis mellifera* (UV: 60% identity; blue: 58% identity; green: 69% identity). The observed homology was 46% between Ha-UV and Ha-BL, 36% between Ha-UV and Ha-LW, and 35% between Ha-BL and Ha-LW.

### 3. Expression levels of opsin genes in various tissues

Before qRT-PCR, the expected PCR products were sequenced for confirmation, and the amplification reactions were validated using standard curve analysis as shown in Figure S2. Table S3 indicates that the expression levels of the three opsin genes were strikingly different according to the tissue in both female and male adults. The highest levels of transcription were observed in the compound eye and brain, with very low transcription levels in other tissues. Thus, compound eyes were collected in the following test to examine the visual function of the opsin genes. In compound eyes, *Ha-LW* exhibited the highest expression level, and *Ha-BL* exhibited the lowest expression level among the three opsin genes (female: *F*_2,6_ = 97.541, *P*<0.001; male: *F*_2,6_ = 471.645, *P*<0.001). The expression levels of the three opsin genes were similar between females and males.

### 4. Diurnal changes in opsin mRNA levels

**Figure 2:**
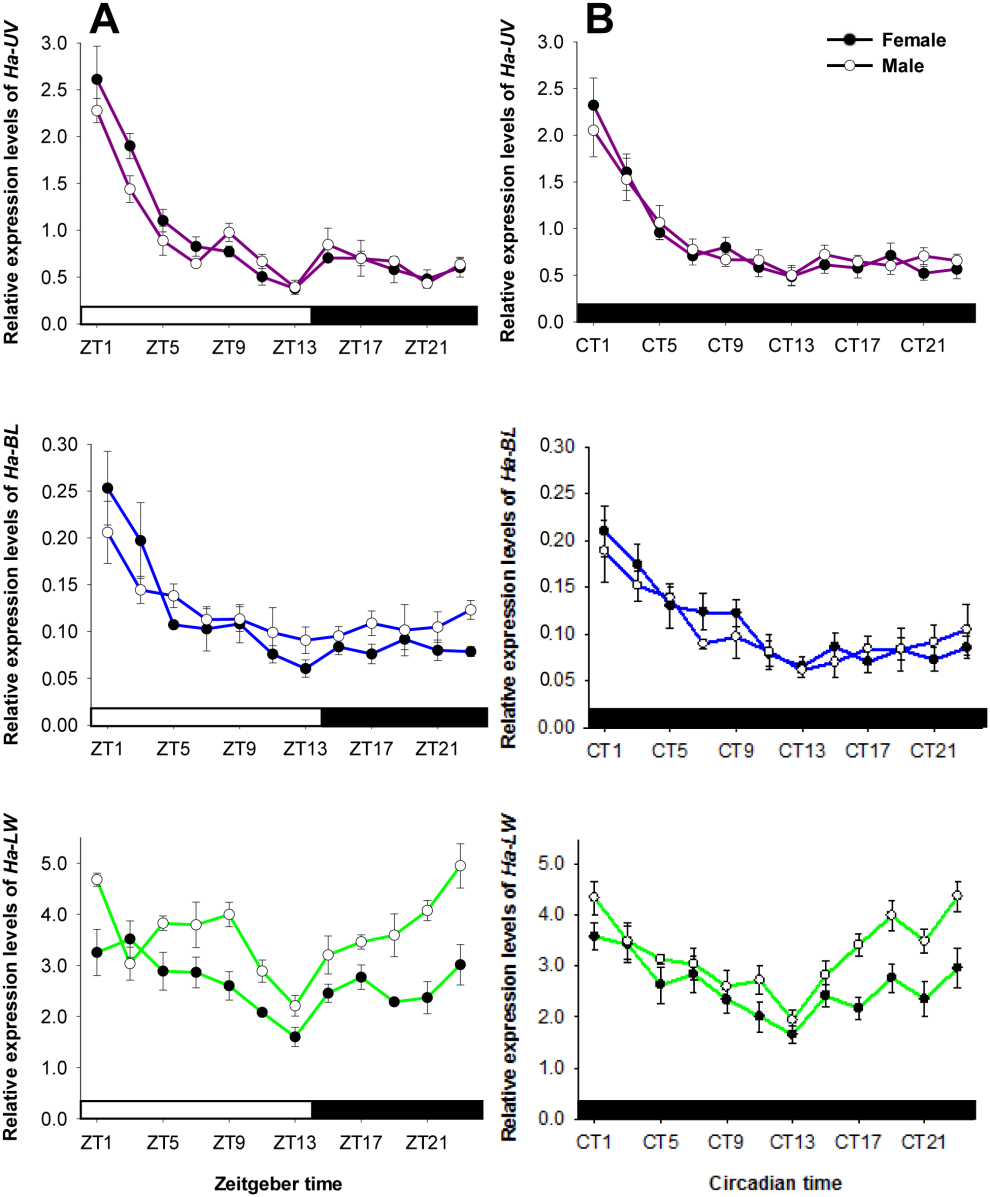
Diurnal changes in opsin gene levels under 14L:10D (A) and DD (B). RNA samples were collected from compound eyes at 2 h intervals. Open bar: day. Filled bar: night. Filled and open circles represent female and male moths, respectively. ZT indicates zeitgeber time; CT indicates circadian time. Each value is the mean ± SE of three collections.

As shown in Figure 2A, *Ha-UV* and *Ha-BL* levels were highest at ZT1 and fell off thereafter (female *Ha-UV*: *F*_11,24_ = 20.522, *P*<0.001; male *Ha-UV*: *F*_11,24_ = 25.046, *P*<0.001; female *Ha-BL*: *F*_11,24_ = 8.177, *P*<0.001; male *Ha-BL*: *F*_11,24_ = 2.945, *P* = 0.013). *Ha-LW* abundance tended to decrease during the day and then increase at night (female: *F*_11,24_ = 3.246, *P* = 0.008; male: *F*_11,24_ = 6.654, *P*<0.001). The expression patterns of *Ha-UV* and *Ha-BL* in females were similar to those in males, whereas the *Ha-LW* levels in females were significantly lower than those in males (for example, ZT1: *t* = 3.036, df = 4, *P* = 0.039; ZT9: *t* = 3.799, df = 4, *P* = 0.019; ZT21: *t* = 4.579, df = 4, *P* = 0.010; ZT23: *t* = 3.300, df = 4, *P* = 0.030). To determine the endogenous characteristics of these oscillations in the compound eye, levels of the three opsin genes were also measured under DD (*H. armigera* was entrained for 1 day under DD). Cycling persisted, exhibiting phases and amplitudes similar to those observed under 14L:10D (female *Ha-UV*: *F*_11,24_ = 16.093, *P*<0.001; male *Ha-UV*: *F*_11,24_ = 10.327, *P*<0.001; female *Ha-BL*: *F*_11,24_ = 6.231, *P*<0.001; male *Ha-BL*: *F*_11,24_ = 4.146, *P* = 0.002; female *Ha-LW*: *F*_11,24_ = 3.511, *P* = 0.005; male *Ha-LW*: *F*_11,24_ = 6.799, *P*<0.001) (Figure 2B).

**Figure 3:**
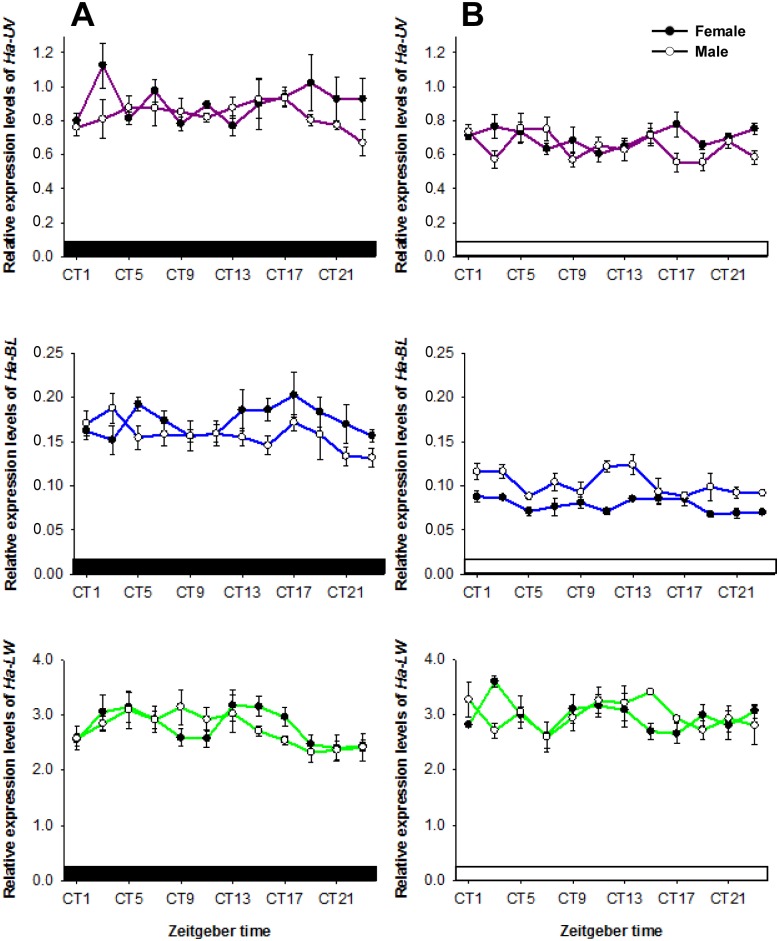
Cycling of opsin mRNA levels ceased under constant DD (A) and LL (B). RNA samples were collected from compound eyes at 2 h intervals. Open bar: day. Filled bar: night. Filled and open circles represent female and male moths, respectively. CT indicates circadian time. Each value is the mean ± SE of three collections.

As shown in Figure 3, no significant changes were observed in opsin gene levels when adults were kept under constant darkness (DD) (female *Ha-UV*: *F*_11,24_ = 1.160, *P* = 0.363; male *Ha-UV*: *F*_11,24_ = 1.068, *P* = 0.425; female *Ha-BL*: *F*_11,24_ = 1.119, *P* = 0.389; male *Ha-BL*: *F*_11,24_ = 1.155, *P* = 0.366; female *Ha-LW*: *F*_11,24_ = 2.165, *P* = 0.055; male *Ha-LW*: *F*_11,24_ = 1.728, *P* = 0.127) or constant light (LL) (female *Ha-UV*: *F*_11,24_ = 1.186, *P* = 0.347; male *Ha-UV*: *F*_11,24_ = 2.083, *P* = 0.064; female *Ha-BL*: *F*_11,24_ = 2.063, *P* = 0.067; male *Ha-BL*: *F*_11,24_ = 2.034, *P* = 0.071; female *Ha-LW*: *F*_11,24_ = 1.707, *P* = 0.132; male *Ha-LW*: *F*_11,24_ = 1.154, *P* = 0.367). Opsin gene levels tended toward upregulation in the transition from constant DD to LL, and *Ha-BL* levels were significantly up-regulated (for example, female ZT5: *t* = 13.755, df = 4, *P*<0.001; ZT13: *t* = 4.206, df = 4, *P* = 0.014; ZT17: *t* = 4.456, df = 4, *P* = 0.011).

**Figure 4:**
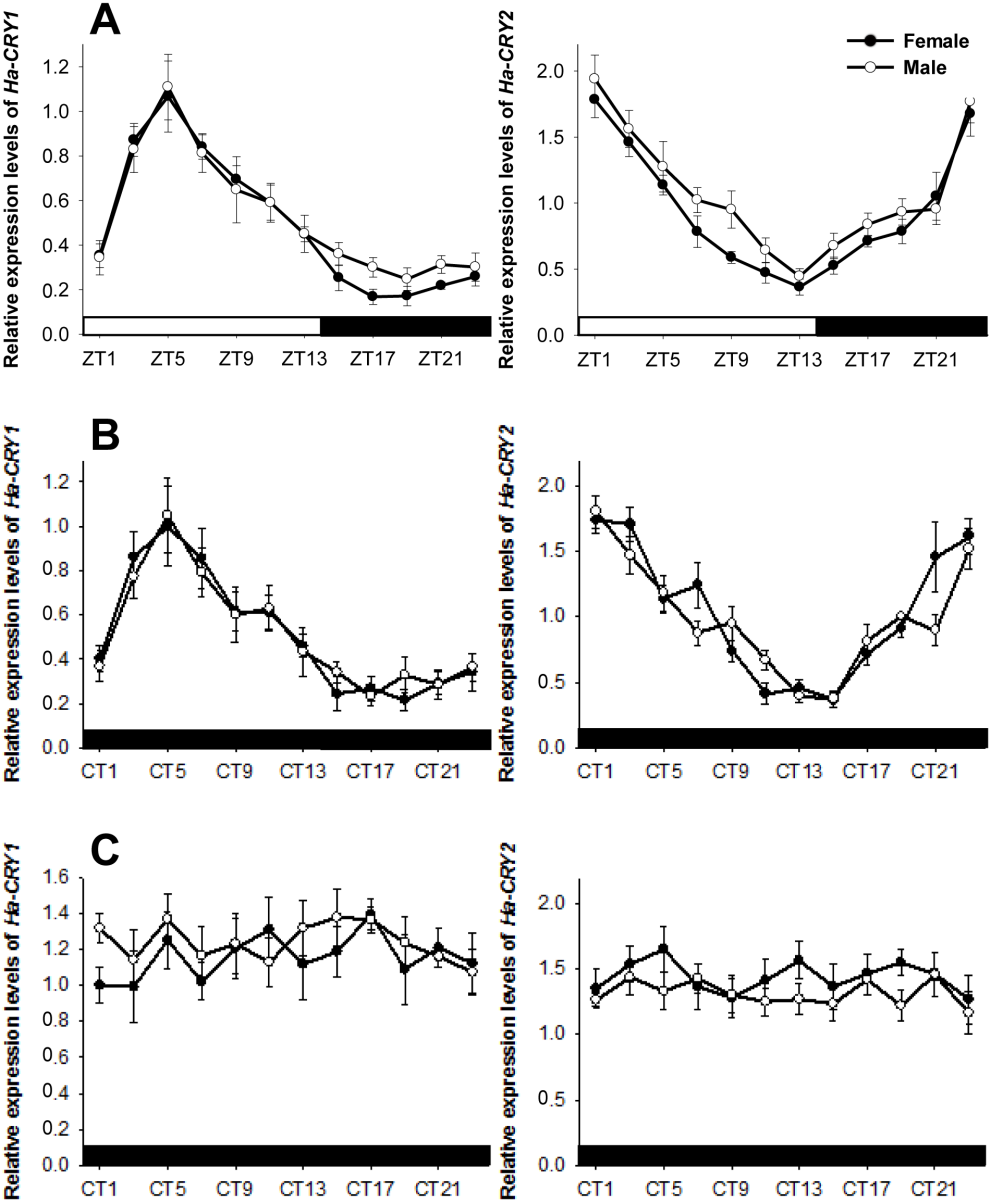
Temporal expression analysis of *Ha-CRY1* and *Ha-CRY2* levels in compound eyes under 14L:10D (A), DD (B), and constant DD (C). RNA samples were collected from compound eyes at 2 h intervals. Open bar: day. Filled bar: night. Filled and open circles represent female and male moths, respectively. ZT indicates zeitgeber time; CT indicates circadian time. Each value is the mean ± SE of three collections.

To determine whether the levels of clock genes oscillated in a circadian manner, RNA was isolated from the compound eyes after *H. armigera* entrainment under standard 14L:10D or DD. Both assays gave identical results: *Ha-CRY1* and *Ha-CRY2* cycled independently, with a peak at ZT5 for *Ha-CRY1* and at ZT1 for *Ha-CRY2* (under LD: female *Ha-CRY1*: *F*_11,24_ = 20.657, *P*<0.001; male *Ha-CRY1*: *F*_11,24_ = 9.466, *P*<0.001; female *Ha-CRY2*: *F*_11,24_ = 19.921, *P*<0.001; male *Ha-CRY2*: *F*_11,24_ = 13.090, *P*<0.001; under DD: female *Ha-CRY1*: *F*_11,24_ = 8.531, *P*<0.001; male *Ha-CRY1*: *F*_11,24_ = 6.279, *P*<0.001; female *Ha-CRY2*: *F*_11,24_ = 16.641, *P*<0.001; male *Ha-CRY2*: *F*_11,24_ = 15.555, *P*<0.001) (Figure 4). However, *Ha-CRY1* and *Ha-CRY2* ceased cycling under constant DD (female *Ha-CRY1*: *F*_11,24_ = 0.645, *P* = 0.774; male *Ha-CRY1*: *F*_11,24_ = 0.636, *P* = 0.781; female *Ha-CRY2*: *F*_11,24_ = 0.579, *P* = 0.827; male *Ha-CRY2*: *F*_11,24_ = 0.743, *P* = 0.689).

### 5. Effects of light exposure on opsin gene expression

**Figure 5:**
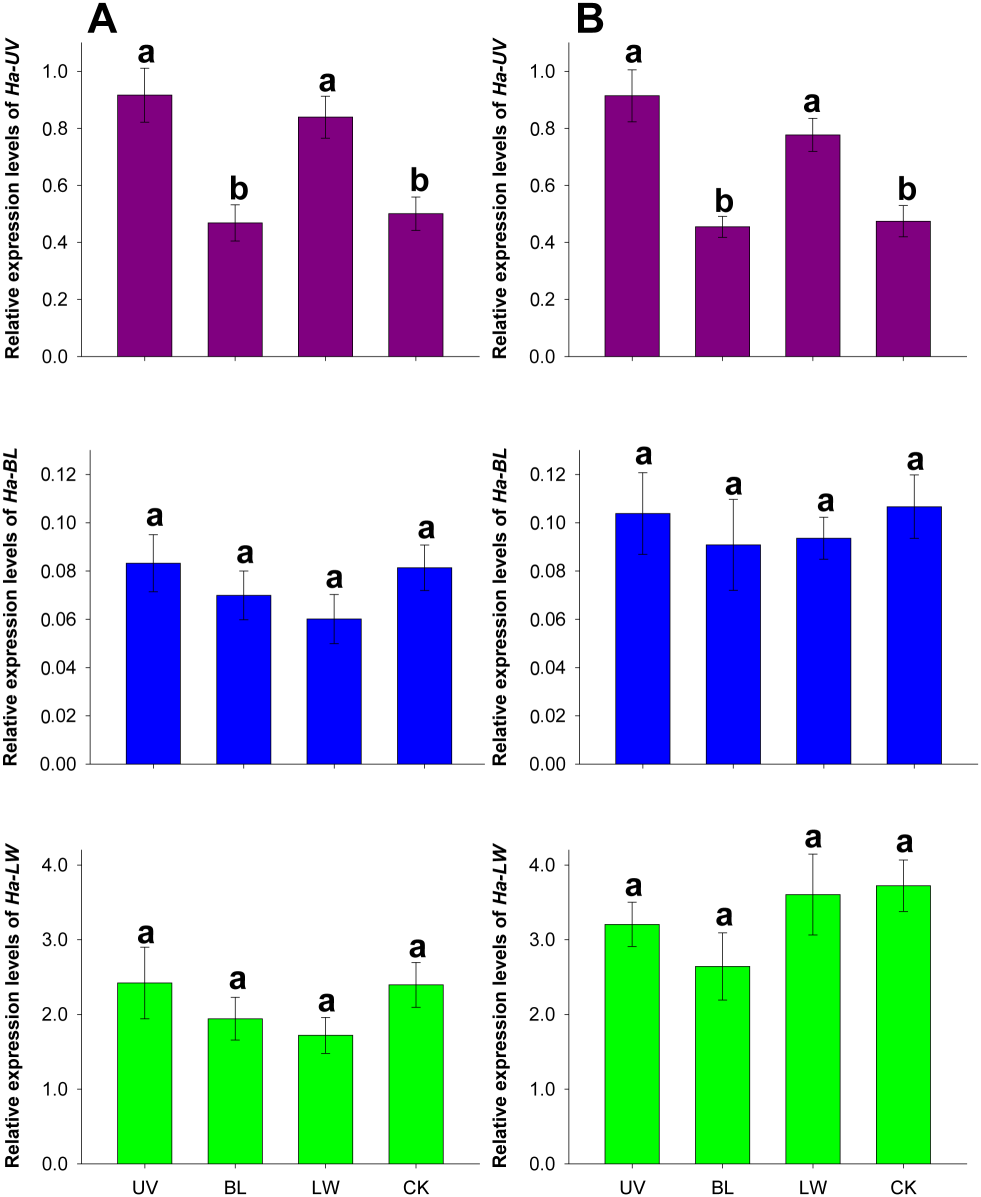
Effects of light exposure on opsin gene expression in female (A) and male (B) moths. RNA samples were collected from the compound eyes of 2-day-old moths after 6 h of UV, blue (BL) or green (LW) light irradiation at ZT20. RNA samples were collected at ZT20 from the compound eyes of moths reared under 14L:10D as CK. Each value is the mean ± SE of three collections. The letters above each bar indicate significant differences according to a Tukey HSD test (*P*<0.05).

As shown in Figure 5, *Ha-UV* levels increased significantly after UV or green (LW) light exposure, whereas *Ha-UV* levels were not up-regulated after 6 h of blue light exposure (female: *F*_3,8_ = 9.620, *P* = 0.005; male: *F*_3,8_ = 12.912, *P* = 0.002). In contrast, light exposure of any wavelength did not significantly up-regulate either *Ha-BL* or *Ha-LW* (*Ha-BL*: female: *F*_3,8_ = 1.067, *P* = 0.416; male: *F*_3,8_ = 0.182, *P* = 0.906; *Ha-LW*: female: *F*_3,8_ = 1.042, *P* = 0.425; male: *F*_3,8_ = 1.355, *P* = 0.324). Light exposure had similar effects on the transcription levels of the opsin genes in both female and male adults.

### 6. Effects of starvation on opsin gene expression

**Figure 6:**
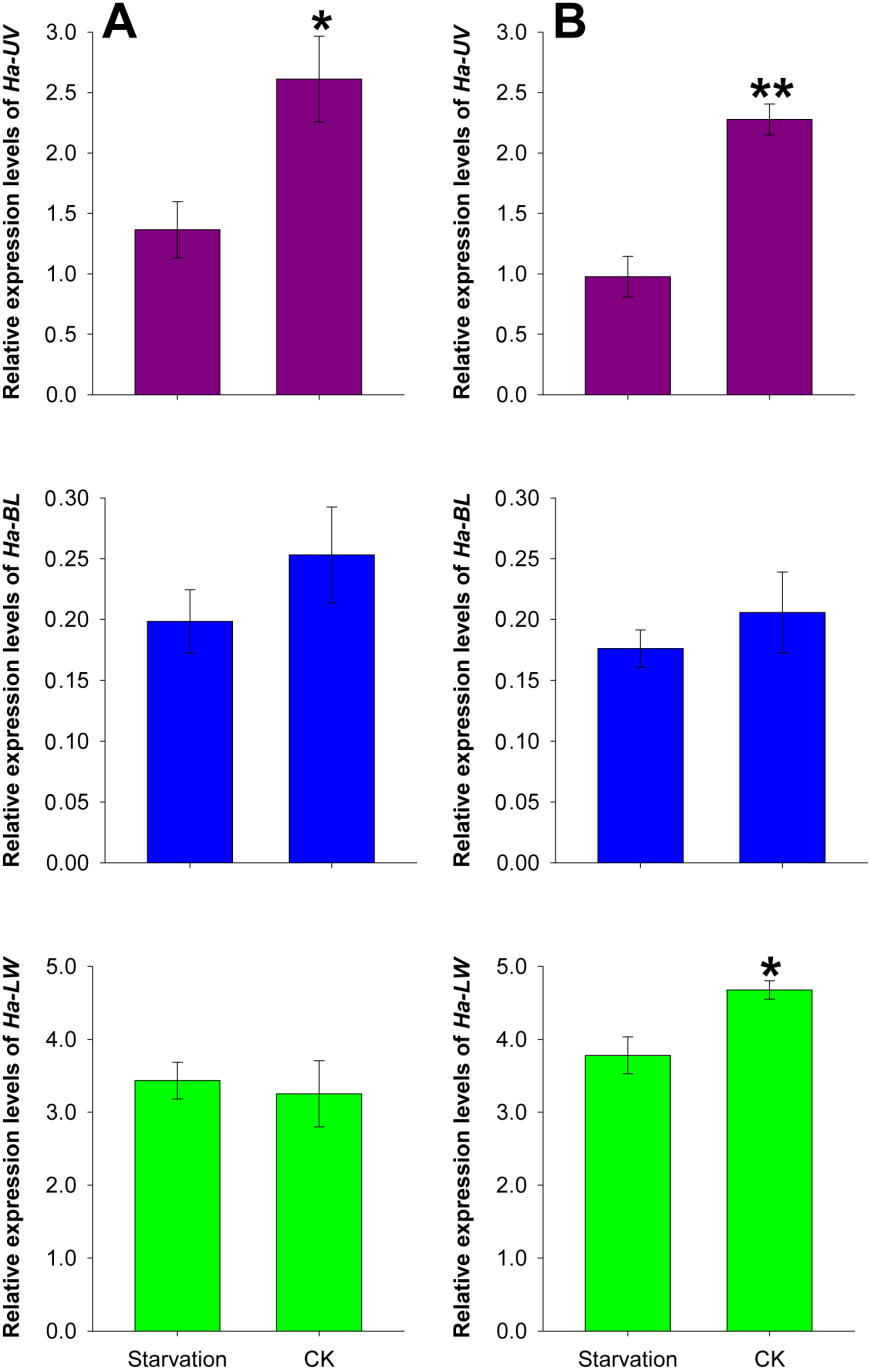
Effects of starvation on opsin gene expression in female (A) and male (B) moths. RNA samples were collected from the compound eyes of 2-day-old moths that were starved after adult emergence at ZT1. RNA samples were collected at ZT1 from the compound eyes of moths reared under 14L:10D as CK. Each value is the mean ± SE of three collections. The “*” and “**” indicate significant differences in opsin gene expression at *P*<0.05 and *P*<0.001, respectively, according to independent t-tests.

Opsin genes tended to be down-regulated following starvation (Figure 6). *Ha-UV* levels significantly decreased after starvation in both female and male adults (female: *t* = 2.941, df = 4, *P* = 0.042; male: *t* = 6.146, df = 4, *P* = 0.004), whereas *Ha-LW* levels were down-regulated only in male adults (*t* = 3.172, df = 4, *P* = 0.034).

### 7. Effects of mating on opsin gene expression

No significant differences in opsin mRNA levels were detected between mated and virgin adults at any time point in either females or males (Figure S3) (for example, *Ha-UV*: 0 h female: *t* = 2.137, df = 4, *P* = 0.099; 0 h male: *t* = 0.994, df = 4, *P* = 0.376; *Ha-BL*: 0 h female: *t* = 0.074, df = 4, *P* = 0.945; 0 h male: *t* = 0.147, df = 4, *P* = 0.890; *Ha-LW*: 0 h female: *t* = 0.535, df = 4, *P* = 0.621; 0 h male: *t* = 1.185, df = 4, *P* = 0.302).

## Discussion

### 1. Correspondence between opsin type and visual pigment in the compound eye

Compared with the methods and primers used in a previous study by Xu *et al.* (2013), we applied new approaches to clone the opsin genes from the cotton bollworm compound eye. Three opsin genes were identified: *Ha-UV*, *Ha-BL* and *Ha-LW*. Based on phylogenetic analysis, the opsin sequences were classified into three main opsin groups, and the alignments indicated high sequence identity among the three opsins (Figure 1). Thus, it is likely that *Ha-UV*, *Ha-BL* and *Ha-LW* serve as receptors for ultraviolet, blue and green light, respectively. Our data confirmed the existence of three spectral photoreceptors in *H. armigera,* which have been identified in the compound eye of *H. armigera* from ERG recordings [39]. All three opsin genes lacked an AATAAA polyadenylation signal. Similarly, a previous study indicated that the UV- and blue-sensitive opsin genes in *Manduca sexta* lack polyadenylation signals [13]. It is likely that the primers annealed to stretches of adenines in the 3′ UTRs of opsin genes rather than to the terminal poly(A) tail.

The *H. armigera* opsins share many characteristics with other members of the opsin superfamily: (1) a Lys in the seventh transmembrane domain that interacts with the chromophore retinal [15]–[17], (2) two conserved Cys residues (Cys127 and Cys204 for Ha-UV, Cys127 and Cys204 for Ha-BL, and Cys131 and Cys208 for Ha-LW) that have been shown to form correct structures in bovine rhodopsin [49]–[50], and (3) two conserved Leu and Asn residues (Leu86 and Asn91 for Ha-UV, Leu86 and Asn91 for Ha-BL, and Leu89 and Asn94 for Ha-LW) that have been demonstrated to be crucial for rhodopsin synthesis during the nascent state [51].

### 2. Opsin genes were highly expressed in the compound eye and brain

The relative expression levels of the opsin genes in various tissues were analyzed to determine a suitable tissue for subsequent tests. The tissue-specific expression data revealed that opsin genes are abundant in the compound eye and brain, with the greatest abundance in the compound eye (Table S3). Opsin gene expression in the brain was previously observed in fish [52]–[53], birds [54], spiders [55] and honeybees [56]–[57]. These results indicated that opsin genes might mediate not only visual function but also nonvisual function. In the current study, compound eyes were collected to examine the visual function of opsin genes. In addition, *Ha-LW* was found to be the most abundant of the three opsin genes. It is possible that *Ha-LW* is important to nocturnal moths because, of the three different wavelength lights, long-wavelength light is the strongest at night. Thus, the elevated expression of *Ha-LW* might be associated with the nocturnal activities of moths.

### 3. Daily changes in opsin mRNA levels were regulated by the circadian clock

Moths feed [58] and navigate [59]–[60] using multiple cues. Visual perception is one of the most familiar forms of stimulus discrimination, and moths require a highly developed visual system. In contrast to butterflies, most moths are primarily nocturnal, meaning that they are active and use dim-light vision at night and rest during the day. Whether opsin mRNA levels oscillate in a circadian manner has been an interesting question for a long time.

In the current study, *Ha-UV* and *Ha-BL* levels peaked at ZT1 and then decreased, whereas *Ha-LW* levels tended to decrease during the day and increase at night (Figure 2A). However, Xu *et al.* [32] examined the expression levels of opsin genes in *H. armigera* and found that diel patterns of opsin mRNA levels did not fluctuate significantly. One possible reason for this discrepancy is that they collected RNA from whole individual moths, which may have obscured the pattern of opsin expression because it was limited to the compound eye and brain. Our result is similar to those of studies in mice [18] and toads [61], in which opsin mRNA levels peak after light onset. These results are in contrast to honeybee, in which long-wavelength-sensitive opsin mRNA levels peak after light onset and then subsequently decrease [22]. Increased *Ha-LW* levels at night might be associated with nocturnal behavior. The cycling of opsin gene expression in *H. armigera* under 14L:10D persisted for one day under DD conditions (Figure 2B) but did not persist further under constant DD or LL (Figure 3), demonstrating that (1) the expression profile of opsin genes depends on an endogenous circadian clock that is independent of light and that (2) opsin mRNA cycling could be disturbed by constant light or darkness. In similar previous studies, opsin mRNA cycling persisted under DD conditions in toads [61] and honeybees [22], and photoreceptors appeared to have an endogenous pacemaker that adjusts the daily opsin mRNA levels.

Clock genes are responsible for synchronizing the endogenous rhythm to environmental conditions. The two clock genes in *H. armigera* (*Ha-CRY1* and *Ha-CRY2*) have been shown to oscillate daily in the head [40]. We determined whether *Ha-CRY1* and *Ha-CRY2* oscillate daily in the compound eye to confirm their circadian function in peripheral tissues. We found that *Ha-CRY1* levels peaked at 5 h after light onset and subsequently decreased, whereas *Ha-CRY2* exhibited an opposite expression pattern (Figure 4). The expression patterns of clock genes in the compound eye of *H. armigera* were consistent with those in the head of *H. armigera* [40] and other species [62]–[64]. We also demonstrated that *Ha-CRY1* and *Ha-CRY2* expression cycling persisted for one day under DD conditions, illustrating that clock genes likely perform circadian functions in the compound eye. The waveform of the *Ha-CRY1* expression pattern was more rounded than that of *Ha-CRY2*, indicating that *Ha-CRY1* might be more sensitive to light. In Lepidoptera species, CRY1 is predominantly a blue-light photoreceptor that entrains the central oscillator, whereas CRY2 acts as a major transcriptional repressor but not as a circadian photoreceptor [40]. Meanwhile, constant DD disturbed *Ha-CRY1* and *Ha-CRY2* cycling, which could be assumed to disturb opsin gene expression patterns.

What is the biological significance of daily changes in opsin mRNA levels? UV and blue light are stronger during the daytime, so increased expression of *Ha-UV* and *Ha-BL* during the day are beneficial for the recognition of short-wavelength light, which may serve to protect moths from UV damage. Moths exhibit limited diurnal behavior and are more active at night. The increase in *Ha-LW* levels at night may be useful in a dim-light environment. Under constant DD, opsin mRNA levels decrease in honeybees [22], whereas the three opsin genes in *H. armigera* increase, suggesting that (1) the mechanism controlling opsin mRNA expression in nocturnal insects differs from that in diurnal insects and that (2) opsin genes may be important for nocturnal activities in long-term darkness. However, we believe that the observed daily changes in opsin mRNA in *H. armigera* did not differ much from those in diurnal insects. In moths, these three opsin genes seem to operate under functional constraints and share conserved functions [32]. Thus, opsin genes in *H. armigera* might play an important role in the perception and discrimination of color.

### 4. Opsin gene levels tended to be up- and down-regulated after light exposure and starvation, respectively

Both light exposure and nutritional status influenced opsin gene expression in *H. armigera*; however, the effects of the two factors differed among the three types of opsin genes. Although opsin mRNA levels were dependent on an endogenous circadian clock, we confirmed that light exposure (UV and green light) rather than the scotophase significantly up-regulated *Ha-UV* (Figure 5). Similarly, previous studies indicated that *OPS1* (*Neurospora* rhodopsin) transcript showed enhanced expression after NUV (mainly 360 nm) irradiation in *Bipolaris oryzae* [29], and UVA (mainly 365 nm) and violet (mainly 410 nm) irradiation induced rhodopsin expression in normal human epidermal keratinocytes [30]. Organisms exposed to more light exhibited enhanced opsin gene expression, and this phenomenon has been observed in *H. armigera* [32], *Apis mellifera* [22], *Ceratosolen solmsi* [31], and *Lucania goodei* [14]. In the current study, light positively regulated the expression of *Ha-UV* in an organism-specific manner, suggesting that light-sensing in *H. armigera* might be important for synchronizing activity with the nocturnal lifestyle. Unlike the effect of light exposure on opsin gene expression, starvation down-regulated most opsin genes, especially *Ha-UV* (Figure 6). Vision plays an important role in locating food, and we initially confirmed that opsin gene levels were influenced by nutritional status. Our study revealed that *Ha-UV* was the opsin gene most affected by environmental conditions (light exposure and nutritional status).

### 5. Sexual dimorphism in opsin gene expression

The rhythmicity of opsin gene expression was not sexually dimorphic, except that *Ha-LW* expression was significantly lower in females than in males (Figure 2). In contrast, in *Poecilia reticulata*, the long-wavelength-sensitive opsin gene is up-regulated in females, allowing them to better discriminate male coloration and courtship displays [33]. Similar to our study, the long-wavelength-sensitive opsin gene in the butterfly *Bicyclus anynana* is up-regulated in males [34], and sexually dimorphic photoreceptors are common in other butterflies [35]–[37]. In numerous species of Lepidoptera, female and male moths produce sex pheromones to induce potential mating partners, and in certain species, sex pheromones play a key role in mating choice and species isolation [65]–[68]. Although opsin gene expression was not influenced by mating (Figure S3), increased expression of *Ha-LW* in males might be important for locating female moths and improving mating success.

## Conclusion

In this paper, we presented a characterization of visual opsins in a nocturnal moth, the cotton bollworm *H. armigera*, which belongs to a markedly early branch within insect lineage. The expression of three types of opsin genes varied according to time of day and environmental conditions and was regulated by a circadian clock, light conditions and nutritional status. *Ha-UV* and *Ha-BL* were more abundant during the day than at night, which aided recognition of short-wavelength light during the day. However, *Ha-LW* levels decreased during the day and increased at night, which might be useful in a dim-light environment. *Ha-LW* levels were up-regulated in males, which might aid in locating potential sex partners and improving mating success over relatively short distances. Interestingly, daily changes in opsin mRNA levels in *H. armigera* did not differ much from those in diurnal insects, suggesting that opsin genes in *H. armigera* might play an important role in color vision, especially color perception and discrimination.

## Supporting Information

Figure S1 **Alignment of three opsin genes isolated as cDNA from the retina of** ***H. armigera*** **.** Residues identical in two or three of the sequences are shown in red. The G-protein-coupled receptor family is boxed. The visual pigment (opsin) retinal binding site is shaded, and the arrow indicates the site of the chromophore Schiff-base linkage.
(TIF)

Figure S2 **Standard curves for the opsin and cryptochrome genes of** ***H. armigera*** **.**
(TIF)

Figure S3 **Effects of mating on opsin gene expression in female (A) and male (B) moths.** RNA samples were collected from the compound eyes of 2-day-old moths that had completed mating 0 h (ZT15) and 3 h (ZT18) before collection. Mean ± SE.
(TIF)

Table S1 **Primers used for gene cloning and qRT-PCR.**
(DOC)

Table S2 **The GenBank accession numbers of the genes used in this study.**
(DOC)

Table S3 **Relative expression levels of opsin genes in various adult tissues.** Means ± SE in columns followed by different letters are significantly different (Tukey HSD test was used to evaluate *HeBL* in females and other comparisons were evaluated using the Games-Howell test, *P*<0.05).
(DOC)
