# Supplementary figures and images for: The Expression of Three Opsin Genes from the Compound Eye of Helicoverpa armigera (Lepidoptera: Noctuidae) Is Regulated by a Circadian Clock, Light Conditions and Nutritional Status
Source: PLoS One. 2014 Oct 29;9(10):e111683. doi: 10.1371/journal.pone.0111683 (PMC4213014; doi:10.1371/journal.pone.0111683)

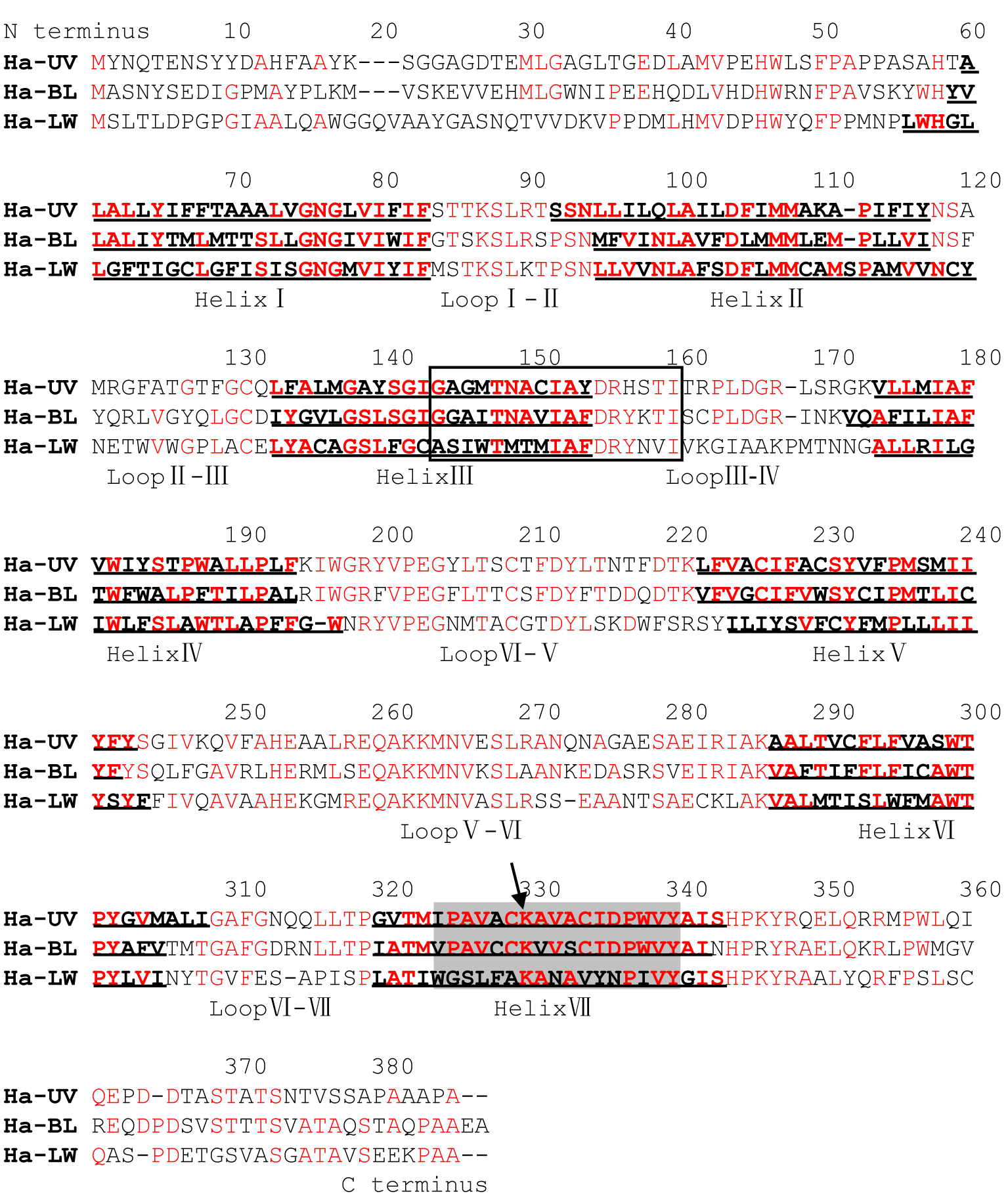

Supplement: Figure S1 — Alignment of three opsin genes isolated as cDNA from the retina of H. armigera . Residues identical in two or three of the sequences are shown in red. The G-protein-coupled receptor family is boxed. The visual pigment (opsin) retinal binding site is shaded, and the arrow indicates the site of the chromophore Schiff-base linkage. (TIF) [file pone.0111683.s001.tif]

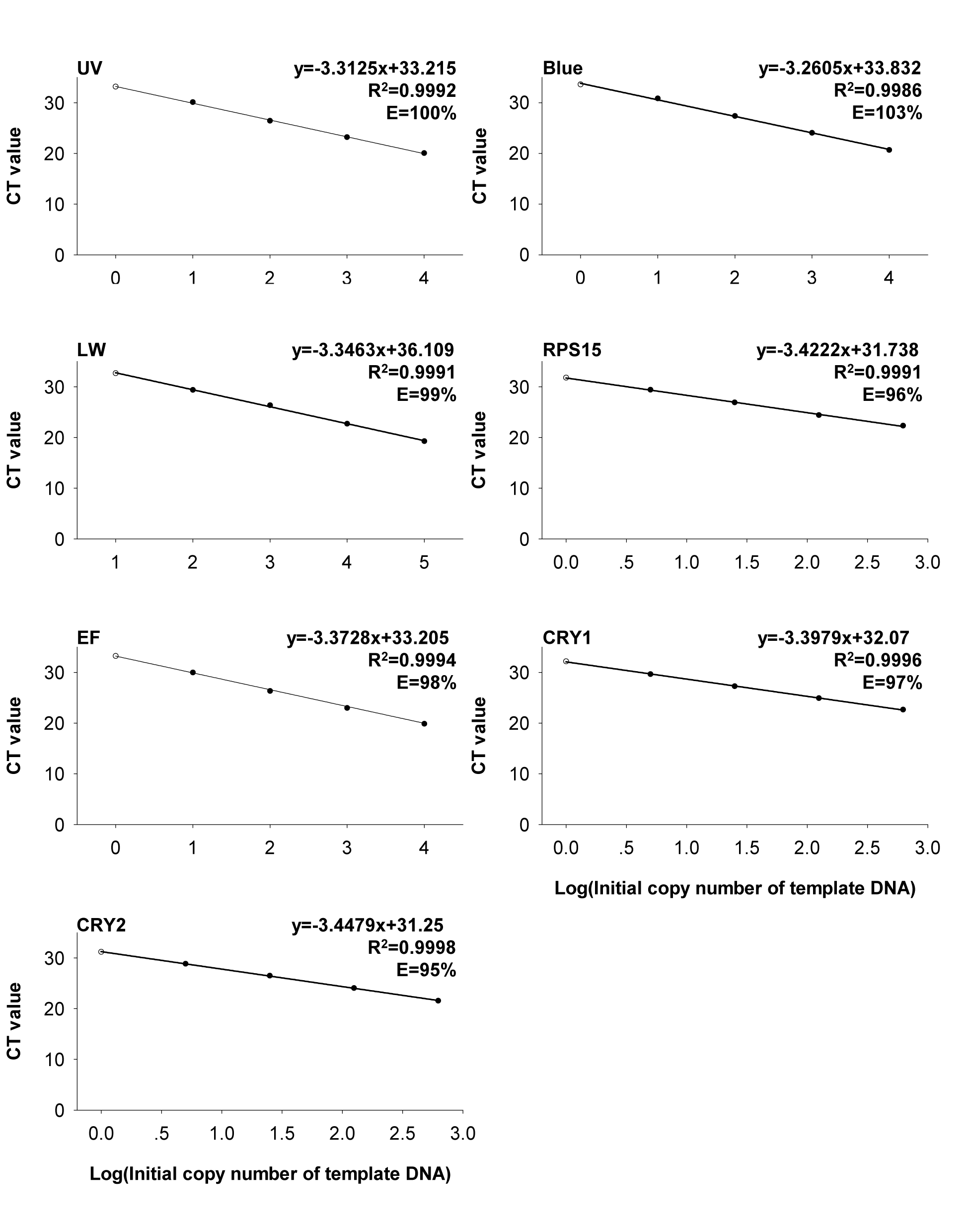

Supplement: Figure S2 — Standard curves for the opsin and cryptochrome genes of H. armigera . (TIF) [file pone.0111683.s002.tif]

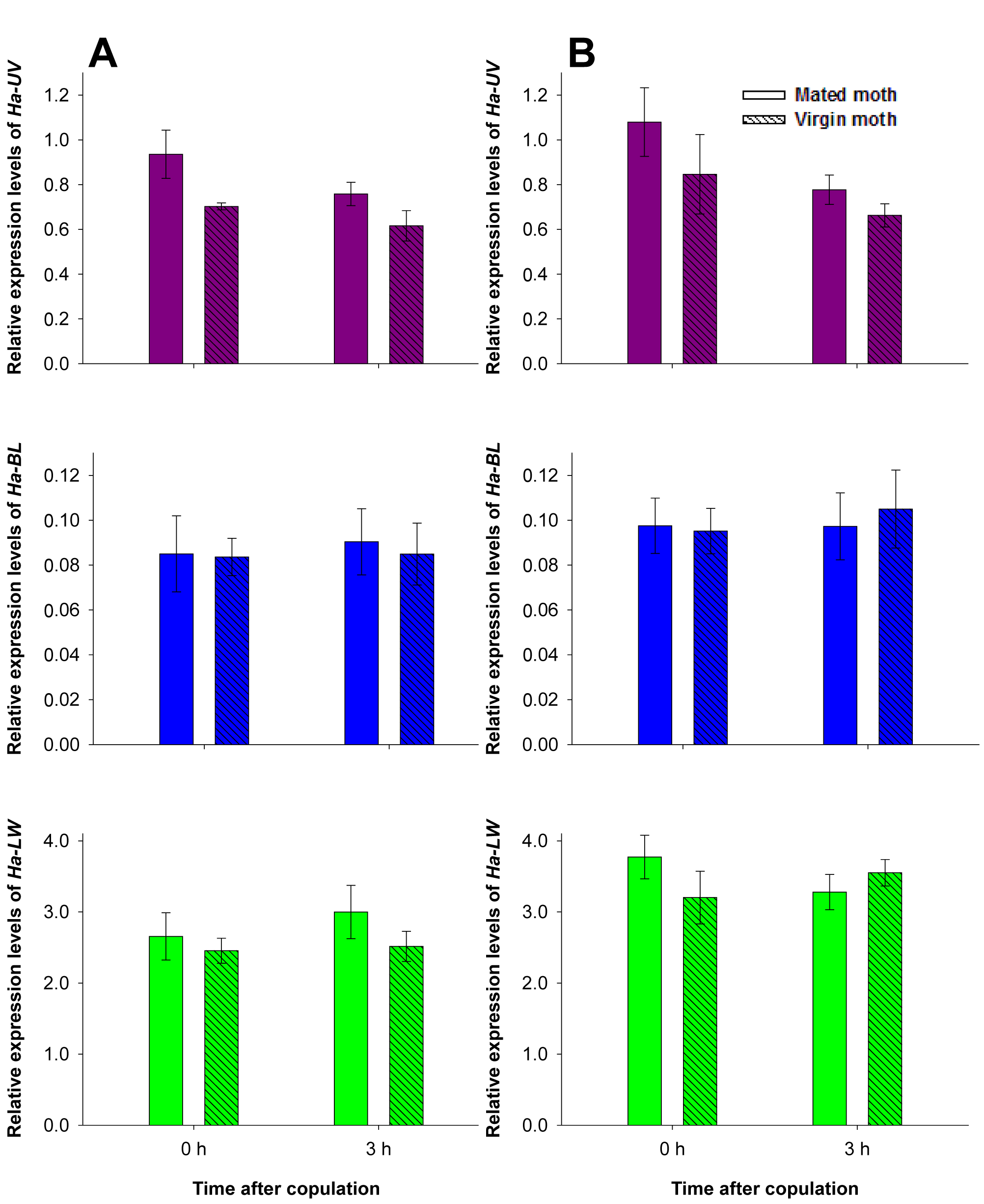

Supplement: Figure S3 — Effects of mating on opsin gene expression in female (A) and male (B) moths. RNA samples were collected from the compound eyes of 2-day-old moths that had completed mating 0 h (ZT15) and 3 h (ZT18) before collection. Mean ± SE. (TIF) [file pone.0111683.s003.tif]
