# Supplementary material for: The Expression of Three Opsin Genes from the Compound Eye of Helicoverpa armigera (Lepidoptera: Noctuidae) Is Regulated by a Circadian Clock, Light Conditions and Nutritional Status
Source: PLoS One. 2014 Oct 29;9(10):e111683. doi: 10.1371/journal.pone.0111683 (PMC4213014; doi:10.1371/journal.pone.0111683)
Supplement: Table S1 — Primers used for gene cloning and qRT-PCR. (DOC) [file pone.0111683.s004.doc]

| Primer name | Sequence 5’ to 3’ |
| --- | --- |
| UVF | GAYTTYATNATGATGGCNAAAG |
| UVR | GGCATNCNNCGTTGNAGTTC |
| BLF | GTNGNACTACTNACTTCGT |
| BLR | CANGTNGTCAGGAANCCCTCT |
| LWF | CAANCNAAAGACTGNAGTAGT |
| LWR | GGNTGGCTGATNCCNTATNCA |
| UV5’ outer-primer | CACCCATGGATCGATGCAAG |
| UV5’ inner-primer | CTTAAAGCGGCTTCATGTGC |
| UV3’ outer-primer | GCATTGGTGCAGGTATGAC |
| UV3’ inner-primer | CCTGAGGGTTATTTGACATCGT |
| BL3’ outer-primer | CACAATGCTGATGACCACCT |
| BL3’ inner-primer | CGCTACTCGTCATCAACTCG |
| LW3’ outer-primer | CACCCGATATGCTTCACATG |
| LW3’ inner-primer | GCTTGCGCTGGCTCTCTA |
| UVQFP | AGCATCGTGGACACCTTATG |
| UVQRP | TGTAGTTCTTGCCTGTATTTCG |
| BLQFP | TGACATATACGGTGTGCTGG |
| BLQRP | GGCAAGATGGTGAATGGTAG |
| LWQFP | GTGCGCGATGTCTCCTGCTATGG |
| LWQRP | CATTGTTGGTCATTGGCTTGG |
| CRY1QFP | CTGCTGGAACGAAACTGGT |
| CRY1QRP | GGAACACGACATTAGGCTTG |
| CRY2QFP | GATCGGAGGAGAAAGTGAAGC |
| CRY2QRP | CGACAAACAGCCAAACCTTAAG |
| EFQFP | GAAGTCAAGTCCGTGGAGATG |
| EFQRP | GACCTGTGCTGTGAAGTCG |
| RPS15QFP | CTGAGGTCGATGAAACTCTC |
| RPS15QRP | CTCCATGAGTTGCTCATTG |
