# Supplementary material for: The Expression of Three Opsin Genes from the Compound Eye of Helicoverpa armigera (Lepidoptera: Noctuidae) Is Regulated by a Circadian Clock, Light Conditions and Nutritional Status
Source: PLoS One. 2014 Oct 29;9(10):e111683. doi: 10.1371/journal.pone.0111683 (PMC4213014; doi:10.1371/journal.pone.0111683)
Supplement: Table S2 — The GenBank accession numbers of the genes used in this study. (DOC) [file pone.0111683.s005.doc]

| Species | UV-sensitive opsin | Blue-sensitive opsin | Long-wavelength-sensitive opsin |
| --- | --- | --- | --- |
| *Helicoverpa armigera* | ADW20311 | AGH28029 | AGH28027 |
| *Agrotis ipsilon* | AEK94178 | AHA48174 | AHA48183 |
| *Manduca sexta* | AAD11965 | AAD11966 | AAD11964 |
| *Macroglossum stellatarum* | AHA48200 | AHA48170 | AHA48188 |
| *Heliconius erato* | AAY16537 | AAY16539 | AAY16540 |
| *Danaus gilippus* | ACC77717 | - | ABY84497 |
| *Lycaena rubidus* | AAT91641 | AAT91640 | AAT91638 |
| *Polyommatus icarus* | ABW69111 | ABD64151 | ABW69110 |
| *Apis mellifera* | AAC13418 | AAC13417 | AAA69069 |
| *Bombus impatiens* | AAV67326 | - | - |
| *Gryllus bimaculatus* | AEG78686 | AEG78685 | AEG78684 |
| *Dianemobius nigrofasciatus* | BAG71429 | BAF45422 | ACN39591 |
| *Acromyrmex echinatior* | EGI64200 | EGI60991 | - |
| *Harpegnathos saltator* | EFN88089 | EFN81524 | - |
| *Apis cerana* | - | BAH04515 | BAH04516 |
| *Nilaparvata lugens* | - | - | BAO03855 |
| *Laodelphax striatella* | - | - | BAO03861 |
