# Supplementary material for: The Expression of Three Opsin Genes from the Compound Eye of Helicoverpa armigera (Lepidoptera: Noctuidae) Is Regulated by a Circadian Clock, Light Conditions and Nutritional Status
Source: PLoS One. 2014 Oct 29;9(10):e111683. doi: 10.1371/journal.pone.0111683 (PMC4213014; doi:10.1371/journal.pone.0111683)
Supplement: Table S3 — Relative expression levels of opsin genes in various adult tissues. Means ± SE in columns followed by different letters are significantly different (Tukey HSD test was used to evaluate HeBL in females and other comparisons were evaluated using the Games-Howell test, P<0.05). (DOC) [file pone.0111683.s006.doc]

| Tissue | | *HeUV* | | *HeBlue* | | *HeLW* | |
| --- | --- | --- | --- | --- | --- | --- | --- |
|  |  | Female | Male | Female | Male | Female | Male |
| Thorax | | - | 14.73e-5±1.71e-5**c** | - | 29.09e-5±5.94e-5**cd** | 15.35e-5±5.33e-5**b** | 14.98e-5±2.06e-5**b** |
| Compound eye | | 2.58±0.15**a** | 2.28±0.13**a** | 25.31e-2±2.20e-2**a** | 17.39e-2±0.61e-2**a** | 3.25±0.23**a** | 4.68±0.13**a** |
| Abdomen | | - | - | - | 63.83e-5±7.26e-5**c** | - | 45.22e-6±2.04e-6**b** |
| Leg | | - | 32.83e-6±7.90e-6**d** | - | 45.92e-6±9.80e-6**d** | 25.25e-6±2.03**b** | 44.33e-6±5.55e-6**b** |
| Wing | | 75.59e-6±9.67 e-6**c** | 61.61e-6±5.23e-6**cd** | 17.41e-6±1.79e-6**b** | 14.91e-6±1.53e-6**d** | 8.00e-5±1.30e-5**b** | - |
| Antennae | | - | - | - | - | 27.72e-6±3.86e-6**b** | - |
| Brain | | 10.84e-2±1.30e-2**b** | 13.41e-2±1.72e-2**b** | 102.41e-4±5.10e-4**b** | 81.39e-4±8.81e-4**b** | 7.14e-1±1.13e-1**b** | 20.34e-2±3.02e-2**b** |
| df | Between groups | 2 | 4 | 2 | 5 | 5 | 4 |
|  | Within groups | 6 | 10 | 6 | 12 | 12 | 10 |
| *F* | | 271.846 | 304.205 | 126.616 | 792.422 | 155.737 | 1.272e3 |
| *P* | | <0.001 | <0.001 | <0.001 | <0.001 | <0.001 | <0.001 |
